# Stakeholders’ Perceptions on the Implementation of the HPV Vaccine School-Entry Requirement in Puerto Rico during the COVID-19 Pandemic

**DOI:** 10.3390/vaccines12070760

**Published:** 2024-07-10

**Authors:** Coralia Vázquez-Otero, Diana T. Medina-Laabes, Derick Pérez-Guzmán, Olga L. Díaz-Miranda, Alondra K. Mercado-Andino, Erika I. Escabí-Wojna, Vivian Colón-López

**Affiliations:** 1 Department for Public Health, College of Health, Community and Policy, University of Texas at San Antonio, San Antonio, TX 78249, USA; 2 Cancer Control and Population Sciences Division, University of Puerto Rico Comprehensive Cancer Center, San Juan, PR 00936, USA; dmedina@cccupr.org (D.T.M.-L.); derick.perez2@upr.edu (D.P.-G.); oldiaz@cccupr.org (O.L.D.-M.); alondra.mercado@upr.edu (A.K.M.-A.); eescabi@cccupr.org (E.I.E.-W.); vivian.colon@upr.edu (V.C.-L.)

**Keywords:** HPV vaccine, HPV vaccine school-entry requirement implementation, COVID-19 pandemic

## Abstract

This study explored the implementation of the human papillomavirus (HPV) vaccine school-entry requirement in Puerto Rico during the COVID-19 pandemic. We conducted 26 semi-structured interviews with stakeholders and community-based organizations from August 2021 to March 2022. The interview guide was developed using the 2009 Consolidated Framework for Implementation Research (CFIR). The interviews were recorded and transcribed in Spanish. Data were analyzed using applied thematic techniques. These themes included the following: (i) Intervention characteristics: Participants noted that the school-entry requirement was effective in increasing vaccination uptake prior to the pandemic. Issues with the immunization registry were noted; (ii) Outer setting: External influences, access barriers, and an increase in HPV vaccine exemptions since the implementation of the COVID-19 vaccine were discussed; (iii) Inner setting: Communication within organizations and HPV vaccination efforts improved as the pandemic progressed; (iv) Characteristics of individuals: Most agreed with the school-entry requirement, including exemptions; and (v) Process: Results showed the need to reinforce the population’s education about HPV and the vaccine. Implementation of the policy was challenging during the early stages of the pandemic due to measures enacted to stop the spread of COVID-19 and focus on the COVID-19 vaccine. Efforts to increase HPV vaccine should focus on increasing HPV vaccine education and creating collaborations.

## 1. Introduction

The human papillomavirus (HPV) vaccine is a safe and effective [1,2] cancer prevention tool that protects against HPV-related cancers such as cervical, oropharyngeal, and anal [3,4]. The HPV vaccine is one of the three strategies proposed by the World Health Organization (WHO) to eliminate cervical cancer as part of their 2020 Global Strategy [5]. The target of this global initiative is to achieve HPV vaccination coverage among 90% of girls between the ages of nine and fifteen by the year 2030 [5]. Puerto Rico (PR) has the highest incidence of cervical cancer of all the US states and territories (12.0 vs. 7.5 per 100,000) [6], and in 2017, had an up-to-date HPV vaccination rate of only 29.26% among 11 to 17 year old girls [7].

According to Law No. 25, enacted on 25 September 1983 (as amended), PR’s Secretary of Health has the authority to determine which vaccines are mandatory for school admission [8]. Thus, as a strategy to increase HPV vaccination rates and decrease cervical cancer rates in PR, the Department of Health (DOH) implemented an HPV vaccine school-entry requirement for 11- to 12-year-old adolescents in 2018. As of 2022, this requirement includes adolescents up to 18 years old [9,10]. The requirement stipulates that students cannot be admitted or enrolled in school if they do not comply with this required immunization, except for those with religious or medical exemptions [8], similar to other United States (US) state’s school-entry requirements [11]. In contrast to Australia [12] and Canada [13], where the HPV vaccine is provided in schools through programs funded by the government, and where uptake is voluntary, in PR, parents must get their children vaccinated at clinics or at their pediatrician’s office [10]. Through the Vaccines for Children Program (VCF), the DOH’s vaccination program offers the HPV vaccine free of charge to physicians contracted under the PR government’s health plan. Additionally, the HPV vaccine is provided to eligible children, including beneficiaries of the government plan, children without health insurance, or those with insurance that does not fully cover the vaccine [14]. For individuals with private insurance, the HPV vaccine is covered.

Barriers and facilitators have been identified in the implementation of the HPV vaccine school-entry requirement policy in PR. Factors at the system–implementer level, including school and clinician nurses sharing their personal experiences to promote the HPV vaccine and the school-entry requirement, as well as community coalitions that educated and trained healthcare providers, have facilitated the process [15]. On the other hand, the cost for private providers to administer the HPV vaccine, social media’s negative influence on parents’ acceptance of the vaccine, and the lack of school nurses have represented implementation challenges for the school-entry requirement [15]. Moreover, parents of unvaccinated children perceived a lack of information about the HPV vaccine and limited communication about the school-entry requirement, posing additional barriers to the implementation of this policy [16].

During the COVID-19 pandemic, vaccine mandates took a toll partly due to the politicization of the discourse surrounding the COVID-19 vaccine [17,18], the proliferation of conspiracy theories by small but vocal groups [19], and the overabundance of misinformation and disinformation that eroded trust in vaccination efforts [20]. Moreover, public health mitigation measures and strategies, such as stay-at-home orders that may have limited access to preventive care services, could have affected HPV vaccination uptake during the pandemic [21]. Thus, this study explored the implementation of the HPV vaccine school-entry requirement in PR during the COVID-19 pandemic.

## 2. Materials and Methods

### 2.1. Overview

The data presented here are part of a five-year study titled “Implementation of School-Entry Policies for Human Papilloma Virus Vaccination (HPV PIVac)” [15,16]. HPV PIVac is a prospective mixed-method study that seeks to understand geographic variation in HPV vaccine policies and outcomes across the US states and territories and examines the implementation of the HPV vaccine school-entry requirement in Puerto Rico. The current analysis uses an exploratory qualitative design and presents data from semi-structured interviews. The HPV PIVac protocol was approved by the Institutional Review Board of the University of Puerto Rico Medical Science Campus (#A8060218) and the Institutional Review Board of the University of Puerto Rico Comprehensive Cancer Center (#2023-04-98).

### 2.2. Participants and Recruitment

Purposive sampling techniques [22,23], including recommendations by the HPV PIVac study’s Community Advisory Board (CAB) and from other key informants, were used to recruit participants. Participants were invited through an official study letter to complete a semi-structured interview either by videoconference (i.e., Zoom) or phone. Eligibility criteria included the following: (1) being older than 21 years old, (2) being able to consent to the recording of the interview, (3) working in an area related to education or health, and (4) having knowledge about the HPV vaccine school-entry requirement. Participants included public and private school system stakeholders, Department of Health (DOH) personnel, healthcare providers, and members of coalitions and community-based organizations (CBOs). The interviews were conducted from August 2021 to March 2022. Interviews were audio-recorded, lasted an average of 25 min (range: 13 to 38 min), and were transcribed verbatim. All participants provided informed consent and received a $30.00 gift card upon completion of the interview.

### 2.3. Procedures

The interviews, conducted in Spanish, were led by team members (RSA, DTML, or ODM) using similar semi-structured interview guides adapted to the type of stakeholder (e.g., school personnel, healthcare providers). The interview guide (Supplementary Materials) included open-ended questions that assessed the stakeholders’ knowledge, attitudes, and perceptions about the HPV vaccine and the barriers and facilitators to implementing the HPV vaccine school-entry requirement, particularly during the COVID-19 pandemic. The interview guide and the codebook, which included input from the CAB, were developed using the 2009 Consolidated Framework for Implementation Research (CFIR). The CFIR is a metatheory that consists of the following five domains: Implementation Characteristics, Outer Setting, Inner Setting, Characteristics of Individuals, and Process [24]. These domains are divided into 39 constructs that target different areas of intervention implementation. Previous research has used this framework to qualitatively study the implementation of the COVID-19 vaccine delivery program in Ontario, Canada [25] and to quantitatively assess changes in HPV vaccine acceptance among healthcare providers in Texas, US [26] during the COVID-19 pandemic.

### 2.4. Data Analysis

The data analysis process was guided by thematic analysis techniques [27], used a codebook based on CFIR, and consisted of multiple iterative steps. First, team members individually coded the interviews, followed by an examination of each quote to achieve codification unanimity, and identification of themes based on the CFIR constructs (CVO, DPG, DTM, and AM). The initial codebook was modified to include additional themes relevant to the implementation as the discussion and coding process evolved. Included themes were evaluated and incorporated under relevant CFIR constructs (e.g., staff—code created as a sub-construct of available resources). Emergent themes were also identified. Regular discussions were held to resolve disagreements or uncertainties and reach consensus as needed. Participants’ quotes were coded using Atlas.ti (v8) [28]. A final review process was performed to ensure that no additional themes emerged, and quotes were allocated to the correct CFIR domain. Data were summarized (CVO, DPG, and AM) into narratives that were reviewed and discussed. During this process, the constructs with limited data (e.g., few participants discussed the topic) or without content were not included. Some of these topic areas are addressed in the limitation section for future research. Following Henninck and colleagues’ approach, descriptive words (e.g., most, some) were used to present the results and highlight the theoretical constructs noted in the data [29]. Lastly, exemplary quotes were translated into English.

## 3. Results

A total of 26 stakeholders participated in this study [23,30]; nine were from the school system, six were employees of the DOH, ten were healthcare providers, and one was a member of a CBO. Below we present our findings, following the CFIR domains and highlighting the impact that the COVID-19 pandemic had on the implementation of the HPV vaccine school-entry policy and on HPV vaccination efforts in PR. See Table 1 for the exemplary quotes.

### 3.1. Intervention Characteristics

This domain included the stakeholders’ perceptions related to the implementation of the HPV vaccine school-entry requirement. They narrated aspects related to the availability of evidence about the HPV vaccine, the effectiveness of the policy, and areas of complexity and cost of implementation.

#### 3.1.1. Evidence Strength and Quality

Most stakeholders agreed that there is sufficient evidence available about the HPV vaccine. Some mentioned the scientific studies as the basis for evidence validity and HPV vaccine effectiveness.

#### 3.1.2. Relative Advantage

Most stakeholders considered that implementing the HPV vaccine school-entry requirement has been an effective strategy to increase the HPV vaccine uptake among adolescents. However, to facilitate the implementation process, some noted that education efforts, such as providing information to providers and parents, in the areas of HPV and the HPV vaccine should continue. The majority of stakeholders agreed on the need for the HPV vaccine.

#### 3.1.3. Complexity

When describing some areas of difficulty with the implementation of the HPV vaccine school-entry requirement, some participants mentioned their concerns with PR’s vaccine registration system (PREIS). These concerns included data entry problems, limited staff to enter the data, and system failure during the pandemic.

#### 3.1.4. Cost

Related to costs associated with implementing the school-entry requirement, some stakeholders focused on several aspects specific to the HPV vaccine. These included the difference in coverage of the vaccine cost, depending on the type of health insurance (i.e., private- or government-sponsored), and the cost for private providers to buy the vaccine and have a special license required by the DOH to keep the vaccine in their offices.

### 3.2. Outer Setting

This domain describes contextual factors including external influences, access barriers during the pandemic, vaccine exceptions, connections with other organizations, and regulations that impacted the implementation of the HPV vaccine school-entry requirement.

#### 3.2.1. Needs and Resources: External Influences

Participants discussed three significant concerns about external influences that hindered successful HPV vaccination during the COVID-19 pandemic. Among these, participants expressed that some parents and religious community members resist the HPV vaccine due to worries about sexual initiation/activity among adolescents at a young age or that the HPV vaccine will cause hormonal changes. The second concern was HPV vaccine misinformation. Participants perceived that by accessing information from unreliable sources such as social networks (e.g., Facebook, YouTube), parents developed distrust towards the vaccine. Most stakeholders did not hold these types of misinformed knowledge or beliefs about the HPV vaccine. The third concern raised was that parents agreed to get their children vaccinated just because it is required by the school, without acknowledging any of the benefits of the HPV vaccine. Despite this, a few stakeholders mentioned that including the HPV vaccine as part of the school-entry requirements facilitated parental recognition of the HPV vaccine’s importance and encouraged them to vaccinate their children.

#### 3.2.2. Needs and Resources: COVID-19 Access Barriers

Most participants reported that community members had limited access to the vaccination centers at the beginning of the pandemic since most clinics were closed due to the lockdown, and few health providers were available. Once the vaccination centers began to open, parents were only looking for the COVID-19 vaccine and not the regular-scheduled, school-entry-required vaccines. Consequently, children’s and adolescents’ HPV vaccine schedules were delayed or not fulfilled. Also, many parents were afraid to take their children to be vaccinated due to the risk of contracting COVID-19.

Some other barriers mentioned by the participants were classes being changed to a virtual modality, the limited number of patients allowed in a clinic, and the focus of all vaccination efforts primarily being on the COVID-19 vaccine. Also, parents having difficulty obtaining the vaccination sheet required by the DOH for school entry and the limited availability of appointments for vaccination services were other barriers identified by the participants. Nevertheless, a few stakeholders reported that following up with parents to complete the HPV vaccine schedule and giving them a sheet with a list of the vaccination centers by municipality facilitated compliance.

#### 3.2.3. Needs and Resources: Exemptions

Participants discussed their experiences with vaccine exemptions. Most interviewees expressed having at least one experience where parents decided not to vaccinate their children, opting for religious or medical exemptions from all the vaccines and/or HPV, but mostly for religious reasons. Stakeholders also affirmed that many religious leaders were certifying these exemptions based on their views, even if their religion did not have an anti-vaccine dogma. Furthermore, naturopaths were also granting and charging a fee for those exemptions. Nonetheless, the DOH must accept these exemptions because they have been notarized. Some expressed having to educate school personnel on managing these exemptions because exemptions due to personal beliefs are illegal in PR. Participants noted that the use of this type of exemption increased during the COVID-19 pandemic, despite its illegality.

#### 3.2.4. Cosmopolitanism

Participant’s organizations often received and used educational materials from institutions such as Merck, the World Health Organization (WHO), and the Centers for Disease Control and Prevention (CDC). At the local level, community-based organizations like the PR Vaccination Coalition (VOCES) provided these materials. In general, healthcare providers were willing to collaborate with schools despite the differences and complexity of the systems (e.g., the PR Department of Education and DOH).

Other collaborations reported included the vaccination health fairs taking place in municipalities’ squares, where mobile immunization clinics were present and community partners were invited to provide education. Receptiveness to these collaborations was reported during the COVID-19 pandemic. For example, television and radio advertising programs to promote vaccines were developed in collaboration with the Puerto Rican Society of Pediatrics. Although most participants reported a positive connection between their organization and external organizations, some noted that their institutions were not networking with others (i.e., the Department of Education) due to a lack of communication from schools seeking orientations and training.

#### 3.2.5. External Policy and Incentives

Some participants discussed that, each year, the DOH releases the school-entry vaccine requirement executive order, including the terms and conditions for vaccine exemptions. While a few stakeholders stated that the information is reaching the parents and the announcement is clear in terms of content, others claimed its superficiality and parental lack of understanding. To disseminate the school-entry requirement letter, some described the use of the school’s webpage as a resource to indicate that every child must have their vaccines up-to-date, and every parent must present evidence of compliance via the vaccination sheet.

### 3.3. Inner Setting

Stakeholders highlighted how the pandemic impacted communication within their organizations, the importance of the implementation of the requirement, the availability of resources, the limited staff, and having access to information as part of the inner setting.

#### 3.3.1. Networks and Communications

Some stakeholders mentioned that the COVID-19 pandemic directly impacted communication within their organizations. They noted that the pandemic transformed and improved their communication by increasing the dissemination of information via email. Interviewees described a positive communication process in which regional and link nurses sent vaccine itineraries for school nurses to revise each student’s record and establish contact with parents whose child did not comply with the requirement. Although only some participants expressed that, before and during the first months of the COVID-19 pandemic, communication within the organization was challenging due to a lack of technological knowledge, as time passed, it became easier to establish direct communication.

#### 3.3.2. Relative Priority

Most participants shared their perceptions of the importance of the HPV vaccine as a requirement for school entrance. However, most mentioned that the COVID-19 vaccine became the main priority when the pandemic started in 2020. Parents and different stakeholders (such as school employees, the PR DOH, and providers from vaccination centers) stopped or postponed the administration and monitoring of regular and school-required vaccines, including the HPV vaccine, to be able to address the efforts toward the public health emergency. However, some stakeholders highlighted the importance of continuing with HPV vaccination efforts and reported concerns about not reinforcing the need for the HPV vaccine. Some participants mentioned that regular vaccination efforts resumed in 2022 and recommended offering the HPV vaccine when parents went to vaccinate their children against COVID-19, facilitating compliance with the HPV vaccine school-entry requirement.

#### 3.3.3. Readiness for Implementation

A few participants indicated organizational commitment to implementing the HPV vaccine school-entry requirement. Most reported being positive and strict in complying with vaccine school-entry requirements. Some healthcare providers mentioned that they have been available since the beginning of the COVID-19 pandemic, have their staff trained, and have always maintained the priority of regular vaccination.

#### 3.3.4. Available Resources

Most stakeholders expressed that their organizations did not encounter any challenges regarding the HPV vaccine supply. Some participants affirmed that there is a need for resources to comply with the process of vaccine requirements, such as orientation and verification of files. This was especially important in schools that were affected by the hurricanes Irma and Maria in 2017, by the series of earthquakes in 2019 and early 2020, or by the COVID-19 pandemic. Regarding access to funding and financial resources, the majority expressed that their organizations did not experience any issues during the pandemic.

#### 3.3.5. Available Resources: Staff

The “Available Resources: Staff” theme was created to include participants’ statements regarding the personnel needed to implement the HPV vaccine school-entry requirement. Most respondents mentioned being understaffed in their respective facilities, including schools and vaccination centers, to implement and follow the policy. The majority reported having only one nurse dedicated to the HPV vaccination, and some of them were also responsible for data entry and other responsibilities, such as doing workshops, orientations, and vaccinating against COVID-19 and other diseases. A few participants reported having sufficient nurses in vaccination centers, but sometimes, providers (i.e., pediatricians) dedicated to vaccination were scarce, which limited the implementation of the HPV vaccine requirement.

#### 3.3.6. Access to Knowledge and Information

Most participants reported having easy access to information about the HPV vaccine and the school-entry requirement within their organizations. The most discussed theme was the education and training their organizations received from stakeholders. Stakeholders narrated receiving visits, training, and educational materials from propagandists and other organizations (e.g., Merck and CDC) that kept their organizations up to date with the latest vaccine information. Participants also noted that their institutions have programs (i.e., quality improvement programs in vaccination services) that concentrate on educating professionals to effectively disseminate information to parents, providers, teachers, and the community. Participants also described obtaining knowledge and information from vaccine-related workshops, conferences, and training about the vaccine school-entry requirement and the immunization platform PREIS. However, due to the complexity of the newly introduced PREIS, a few participants reported still having doubts about the platform. While some stakeholders were optimistic about these efforts, a few felt that efforts had diminished and did not cover HPV-related themes due to the COVID-19 pandemic.

### 3.4. Characteristics of Individuals

This domain describes the stakeholders’ beliefs, knowledge regarding vaccine exemptions, and their ability to implement the HPV vaccine school-entry requirement.

#### 3.4.1. Knowledge and Beliefs: Exemptions

Participants shared their opinions on the vaccine exemptions. Most reported knowing about the medical and religious exemptions established under PR’s immunization law. Some mentioned respecting these exemptions as it is a legal right. However, a few other participants considered only the medical exemptions as valid and some participants reported that religious exemptions should be eliminated because no religion in PR prohibits vaccination.

#### 3.4.2. Self-Efficacy

Some participants recognized their abilities to implement the HPV vaccine school-entry requirement during the pandemic. Among these were verifying the students’ vaccination sheet for compliance with the requirement according to age and grade and coordinating vaccination appointments. Some participants mentioned creating educational materials about the HPV vaccine, health, and safety aspects. These materials offered information about the school-entry requirement as a preventive measure, specially directed to parents who did not want to vaccinate their children, students, and the general community.

### 3.5. Process

To actively implement the policy, stakeholders described strategies that facilitated implementation, such as education and promotion efforts. They also reflected on their experience implementing the HPV vaccine school-entry requirement during the pandemic, highlighting the need for collaborations.

#### 3.5.1. Engaging

Most participants commented about strategies that facilitated the implementation of the HPV vaccine school-entry requirement. The most mentioned strategy was educating parents, healthcare providers, school employees, students, and the general population about the HPV vaccine. Some approaches that facilitated the HPV vaccine uptake through education efforts included showing images of how HPV manifests in the body through pictures of genital warts, providing literature about the different types of cancer that HPV could cause if not vaccinated, and explaining the benefits of the HPV vaccine to parents who were reluctant to vaccinate their children. The second strategy mentioned was contacting parents by making phone calls, sending e-mails and texts, sending letters reminding and following up about vaccination appointments. The third strategy described was the promotion of the HPV vaccine through TV commercials and social media with educational information. Moreover, a few participants mentioned taking advantage of the moment when the minors are getting other vaccines to offer the HPV vaccine.

#### 3.5.2. Reflecting and Evaluating

Most participants offered recommendations regarding their experience with the HPV vaccine school-entry implementation during the COVID-19 pandemic. The majority highlighted the need to provide and reinforce education about the effectiveness, importance, and benefits of the HPV vaccine and the school-entry requirement. Stakeholders mentioned some promotion strategies to disseminate information about the HPV vaccine, such as TV, newspaper, and social media ads; press releases; phone lines for orientation; eye-catching infographics; and developing a circular letter of the school vaccination requirement in a more straightforward language for the general community. Other strategies recommended included offering workshops for school nurses and non-clinical staff and educating young people so that they could promote the message to their peers and others. Some participants reported the need for collaborations between external organizations, such as vaccination clinics, schools, and the general community to continue educating and facilitating access to HPV vaccine appointments.

## 4. Discussion

During the COVID-19 pandemic, the implementation of vaccine mandates may have been interrupted due to the public health measures taken [21], the politicization of the discourse around vaccines [17], and the misinformation and disinformation that eroded trust in vaccination efforts [20]. Guided by CFIR, this study explored the implementation of the HPV vaccine school-entry requirement in PR during the COVID-19 pandemic. Our findings show how there were barriers and facilitators that affected the continuation of the implementation of the HPV vaccine school-entry requirement in PR during the COVID-19 pandemic that should be noted.

As described by the participants, the HPV vaccine school-entry requirement facilitated HPV vaccination efforts in PR by encouraging hesitant parents to get their children vaccinated. However, implementing this policy was challenging during the early stages of the pandemic. This was due, in part, to public health measures enacted to stop the spread of COVID-19. Similar to findings from Ryan and colleagues [21], in a rural midwestern US state, barriers to HPV vaccination uptake included issues with the closing of clinics and patient concerns about COVID-19 infection. Moreover, once the COVID-19 vaccine became available in PR, public health vaccination priorities shifted, allocating resources, the already limited staff, and more time to the COVID-19 vaccination efforts.

Health misinformation related to vaccines has been found to be common on social media platforms such as WhatsApp and Facebook [31]. For instance, Kearney and colleagues (2019) found that anti-vaccine posts on Instagram frequently presented deceptive information about the HPV vaccine [32]. Additionally, false information about COVID-19 on social media posts has been reported as part of a systematic review [33]. The COVID-19 infodemic [34], which included an abundance of misinformation, affected vaccination efforts globally [20]. In PR, continued HPV vaccine misinformation and disinformation on social media and shared through social networks amplified during the COVID-19 pandemic. This resulted in a mistrust of vaccination efforts by the government. Moreover, common myths about the HPV vaccine [35,36], such as the belief spread by religious groups that it initiates sexual activity, continue to cause hesitancy among parents. For example, stakeholders noted that during the COVID-19 pandemic, vaccination exceptions mainly based on personal beliefs increased, despite these being illegal in PR. These factors, coupled with issues regarding PR’s vaccine registry, limited staff, providers’ costs associated with buying the HPV vaccine and keeping their license, and shifts in public health priorities, made the implementation of the policy challenging.

Despite these barriers, efforts to increase HPV vaccination rates should continue. These efforts should include providing education and information about the HPV vaccine and the HPV vaccine school-entry requirement to parents, providers, school employees and students, and the general population [37,38]. Recommendations to continue education efforts about the HPV vaccine have been reported for other countries such as Poland, Indonesia, and Saudi Arabia [39,40,41]. As recommended by the stakeholders, this information could include clarifying the HPV vaccine school-entry requirement for parents using an announcement to provide further information about the HPV vaccine at schools and school websites. Social media platforms could be used as another tool to communicate correct information about the HPV vaccine [42] and the HPV vaccine school-entry requirement.

Collaborations during the COVID-19 pandemic were helpful in promoting vaccination and spreading correct HPV vaccine information. Although communication was cumbersome at the beginning, the increased use of technology during the pandemic facilitated communication and collaboration within and between organizations. For instance, collaborations between state and local health departments to improve vaccination rates [43], as well as partnerships between clinics and community organizations, have been suggested to improve communication and achieve common health goals through difficult times, such as during the COVID-19 pandemic [44]. It is worth noting that engaging with the school system should be promoted to not only keep track of vaccination rates but also provide vaccine information to parents, school nurses, and staff to facilitate the implementation of the HPV vaccine school-entry requirement.

There are some limitations that should be noted. Due to the qualitative nature of this study, findings must be considered within the context of the time and place the data were collected. Thus, generalization is limited. In our analysis, some of the CFIR constructs had no or limited data. This might have happened due to participants in our sample not having the expertise or experience in those areas, or that during the pandemic those constructs were not relevant. For instance, some constructs relevant to the HPV vaccine school-entry requirement that should be further studied due to our limited data include adaptability, learning climate, and leadership engagement. Future research should explore these constructs and include participants from other expertise areas. Moreover, the perspectives of students and parents as recipients of this policy intervention should also be explored.

## 5. Conclusions

Despite barriers confronted during the COVID-19 pandemic, such as the measures taken to decrease the spread of COVID-19 and the focus on the COVID-19 vaccine, the implementation of the HPV vaccine school-entry requirement in PR continued. Efforts to increase knowledge about the HPV vaccine and the school-entry requirement should provide information to parents, increase HPV vaccine promotion, and create community collaborations to support the implementation of this policy. This approach will help eliminate cervical cancer and other HPV-related cancers in the Puerto Rican population.

## Figures and Tables

**Table 1:** Exemplary quotes for factors influencing the HPV vaccine school-entry requirement in Puerto Rico by CFIR domains and for additional themes.

Domain	Construct	Sample Quote
Intervention Characteristics ^a^		
	Evidence strength and quality	*‘I think that there is enough* *research at the global, national, and local level, although probably not laboratory based at the local level, as we know as phase 1 or phase 2; but we have plenty of research that goes beyond, not only clinical investigation or basic science but also epidemiological or behavioral studies that provide us much information about the use of the vaccine’.—* *48*
	Relative advantage	*‘When it was not a requirement [HPV vaccine] they [parents] did not want to vaccinate them [children], you could talk to them, told them about the advantages, and they would answer “no, that is not a requirement”. Once it was made a requirement, acceptance of the vaccine has improved’.—52*
	Complexity	*‘I’m the only nurse for this school enrollment and I have been for 20 years, and I have always submitted vaccine reports and submitted reports for Law 63, and for everything that comes up here and there little by little. But I have to tell you that it has not been the same this year. For the first time in 20 years, this is not the same. Because the vaccination report system, for example, the PREIS, uh, uh, the person who ma- whoever sat down to complete that report, that program, uh, sat down thinking that there was only one employee to do that. […] I don’t know what institution has just one employee, to enter vaccines only. Because they ask for a lot of information and the program is not friendly. At all. It’s very different to PRIS. PRIS was more user friendly’.—68*
	Cost	*‘Well, a barrier is that we are a private clinic; therefore, we do not work with the government plan. Another barrier would be the cost of the vaccine because some do not have health insurance and cannot afford it’.—71*
2. Outer Setting ^b^		
	Needs and resources: external Influences	*‘… they [parents] are really afraid that there will be hormonal changes or that the student will have more sexual desire before the age that is supposed to’.—58* *‘There is always misinformation; parents have told me, “I saw a video on YouTube that some children died from the papilloma vaccine, or it left them with sequelae”’.—66* *‘… many parents have said, if it is not mandatory [HPV vaccine], I am not going to give my child the vaccine’.—66*
	Needs and Resources: COVID-19 access barriers *	*‘At the time of the lockdown, there were barriers because the providers were not available, not because the Department of Health caused them [the barriers], the pandemic itself did. Now, the children are going back to school, and I understand that now is when they will catch up [with the HPV vaccine schedule]… Of course, the COVID pandemic limited the administration of the vaccine’.—72* *‘At first, many people stopped going out due to fear when the lockdown came because everything was closed, and we complied with the government’s order. Once everything started to open, people were still afraid of COVID-19. Then, when the [COVID-29] vaccine came out, many people have been looking for the COVID vaccine and have forgotten about the other vaccines’.—66*
	Needs and resources: exemptions *	*‘I have students who have brought me medical or religious exemptions. I have also had students who have brought them for years and for now have decided to get the vaccines’.—59* *‘Every week, I get one or two exemptions that the schools share with me because they have doubts about whether they comply with the document, so they are not vaccinated against the HPV because it can be a reluctance to any vaccine. However, it [the exemption] almost always arrives due to the HPV...’.—47*
	Cosmopolitanism	*‘Orientation is given to providers when there is new information or workshops. Annually, communication is maintained with the school nurses… If we receive calls from the educational institutions, nurses, staff, or providers, we guide them’.—49* *‘This weekend, I received an email about an advertising agency that is going to give certain programs on vaccination. I don’t know if HPV specifically, but they are always mentioned, and certain colleagues are invited to participate in radio and television programs, so we are always aware of continuing to guide the community about the importance of vaccination’.—57*
	External policy and incentives	*‘For me it is very superficial [the official letter]. The topic of papilloma should be covered more because there are many issues about the vaccine. The parents should be more informed so that they have the correct information because they get carried away a lot by erroneous and unreliable sources’.—56*
3. Inner Setting ^c^		
	Networks and communications	*‘It has improved a lot… Before, you had schools that were not necessarily as technologically advanced, emails were not used. The communications to the parents were sent by letter with the child... Those who had not reached this level of technology, had to reach this point, and now it is much easier to communicate’.—64*
	Relative priority	*‘... the institution gives more priority to COVID than to regular vaccines. It has been tough, I have vaccines that are about to expire, and they even closed the Vaccine for Children area to me because I have to vaccinate for COVID. We have less space to vaccinate now, less time for regular vaccination and more time for COVID’.—63* *‘... my biggest concern is that now we only talk about COVID, and we are failing to reinforce vaccination for all other vaccines’.—64* *‘... if the patient is going to get the vaccine against COVID, a reminder could be given that these other vaccines are important’.—57*
	Readiness for implementation	*‘We kept vaccinating all the time. The fact that the parents did not arrive is another thing, but here we were available and never closed. Even the Health Department webpage went down, and we did the work manually; the data entry was done’.—69*
	Available resources	*‘... Since 2017 when Irma and María [hurricanes] came, the list of priorities was distorted because we were without power for three months and we had a situation to resolve in order to continue with the educational process in those circumstances. Once we recovered from that and we were trying to return to normality, the earthquakes came. We had to make rectifications... Since the wheel has kept changing for the past three or four years, a new priority appeared that jumped on top of us and we did not have the time to be able to control all these aspects’.—64*
	Available resources: staff *	*‘When it was back to school, the supervisor was able to get me someone who was in COVID, to give vaccines and tuberculin [test] because the problem is that I not only vaccinate, I also do tuberculin [test]. Sometimes I had 10, 15, and 20 tuberculin’s tests in one day, which was a tough overload. I had an overload, really a lot’.—70*
	Access to knowledge and information	*‘In terms of a specific vaccine, it has been long, maybe about four or five years that we do not receive a training as part of the Department of Health based on what the HPV vaccine specifically is’.—66*
4. Characteristics of Individuals ^d^		
	Knowledge and beliefs: exemptions *	*‘I understand that there are very few patients who are really justified [exemptions]. I recognize that each parent has the right to choose what is best for their children, and in that sense, we try to educate them so they can make an informed decision. I understand that the exemptions have to be very limited to have the population vaccinated and protected from different diseases, including HPV’.—62* *‘I think the religious exemption is wrong because here, there is no religion that prohibits the vaccine, but parents look for any pastor and sign it for them, and no pastor or lawyer knows more than a clinician. I only trust a clinical specialist who knows the minor’s health condition’.—63*
	Self-efficacy	*‘… I check everything up to the eighth graders, and if there is someone who does not meet the requirement, we follow up, and the parents are called to notify them’. –50* *‘I send the requirements of the school year to all the educational institutions to explain the requirements. The HPV vaccine is required to enter school with at least a single dose. We give orientations to public and private schools, universities, and vaccination providers’.—54* *‘If they do not comply with the requirements, I give orientation to the parents through an informative document that I have generated, and I give follow-up through phone calls’. –68*
5. Process ^e^		
	Engaging	*‘Providers are taught strategies for the follow-up of these patients, to capture them since before the vaccination appointment, they are already sending them to notice either by regular messaging* via *cell phone, electronic system, phone calls, and little cards designed to send as reminders’.—47* *‘…the VOCES coalition and the Health Department went on television programs to educate the community and reinforce the children’s vaccination’.—57* *‘…if you have a student there with some required vaccines due, take advantage, and give them the human papillomavirus. Obviously, education must go hand in hand; the health professional must explain to the mom, dad, or tutor why vaccinating is important’.—48*
	Reflecting and evaluating	*‘I believe efforts must continue to educate at the level of school’ directors and nurses. I know that the Department of Education has done that and has done different programs with grants, but I think that giving more tools at the school level is essential to implement it [the requirement]’.—57* *‘The Department of Health could offer more promotion on television or in the press so that parents can be oriented and go to vaccinate their children’.—54* *‘In terms of schools, continue carrying the message of the importance of vaccination, so that the students, in turn, carry it to their parents because on many occasions, if the parent is a little reluctant, the young person can carry the message’.—60* *‘… To the extent that you can solve some of these problems for the parents… you don’t have to keep reaching them, just take the vaccination center to the school’.—64*

Notes: The following constructs were not induced due to limited or no content: ^a^ Intervention characteristics constructs: intervention source, adaptability, trialability, and design quality and packaging. ^b^ Outer setting constructs: peer pressure. ^c^ Inner setting constructs: structural characteristics, culture, implementation climate, tension for change, compatibility, organizational incentives and rewards, goals and feedback, learning climate, and leadership engagement. ^d^ Characteristics of individuals constructs: individual stage of change, individual identification with organization, and other personal attributes. ^e^ Process constructs: planning, opinion leaders, formally appointed internal implementation leaders, champions, external change agents, and executing. * Additional code created because of its relevance to the implementation.

## Data Availability

The raw data supporting the conclusions of this article will be made available by the authors on request. The data are not publicly available due to privacy or ethical restrictions.

## Supplementary Materials

The following supporting information can be downloaded at: https://www.mdpi.com/article/10.3390/vaccines12070760/s1, The interview guide.
