# Supplementary material for: Stakeholders’ Perceptions on the Implementation of the HPV Vaccine School-Entry Requirement in Puerto Rico during the COVID-19 Pandemic
Source: Vaccines (Basel). 2024 Jul 10;12(7):760. doi: 10.3390/vaccines12070760 (PMC11281630; doi:10.3390/vaccines12070760)
Supplement: Supplementary file 1 [file vaccines-12-00760-s001.zip › vaccines-3068111-supplementary.pdf]

UNIVERSITY OF PUERTO RICO
COMPREHENSIVE CANCER CENTER
POPULATION SCIENCES AND CANCER CONTROL

KEY INFORMANT INTERVIEW – CONSOLIDATED ENGLISH VERSION

Implementation of School-Entry Policies for Human Papillomavirus Vaccination

HPV PIVac
COMPREHENSIVE CANCER CENTER UPR

In collaboration with: Community Advisory Board-HPV PIVac and Dr. Pamela Hull, Prof. University of Kentucky Markey Cancer Center Lexington.

Key Informant Interview Questionnaire

Date: ___/___/___

Time: _____/_____

Administered by: ___________________

ID of Key Informant: ________________________

Position: ____

Type of Organization: ________________________

Interviewer: Please read the following information to the participants:

Thank you for taking the time to speak with us regarding your role in HPV immunization in Puerto Rico. Each section of the interview will be moderated by a member of the research team and will last approximately 60 to 90 minutes. Your personal information or identifiers will not appear in any recording or written material of the study. The research team will create a fictitious identifier for each participant so that your actual name will not be linked to any data contained in the digital recordings.

In this key informant interview, we will conduct a semi-structured and conversational interview to obtain a framework of the barriers and opportunities faced in policy during the early years of implementation. We will use the Consolidated Framework for Implementation Research (CFIR) to ensure we are sufficiently inclusive.

The following table contains the questions to be discussed during the interview, organized by CFIR domain and selected constructs from the domains:

Table 1. Examples of domains according to the Consolidated Framework for Implementation Research (CFIR)

Domains	Description
Characteristics of the intervention	Current practices for HPV vaccination, advantages and disadvantages of the proposed study over current practice.
External context	Perceived needs for HPV vaccination and immunization services (general) in the community.
Internal context	Perception of leadership, commitment, priority of HPV vaccination, current workflow, and team resources.
Individual characteristics of providers	Knowledge, attitudes, and beliefs about HPV vaccination, self-efficacy to offer HPV vaccination.
Process	Planning, level of commitment of implementers, evaluation.

Interviewer: Please ask the following questions:

1. **Could you indicate which organization you work for and provide a brief description of the organization?**
    Probe: What is your position? Years of experience.
    Probe: What is your role in implementing the HPV vaccine requirement for school entry?

2. **What do you know about the Human Papillomavirus (HPV) vaccine?**
    Probe: What is your opinion about the HPV vaccine? What are the pros and cons?
    Probe: Do you believe there is sufficient scientific evidence that the vaccine is effective?
        Probe: If you believe it is effective, what types of cancers does it prevent?
    Probe: Is there any information about the vaccine that makes you hesitant to administer it?
    Probe: Is there any information about HPV you would like to know?

3. **What do you know about the HPV vaccination requirement in schools?**
    Probe: Do you know the ages at which this school vaccine requirement is requested?
    Probe: How many doses of the HPV vaccine are required?
    Probe: Do you believe this HPV vaccine requirement in schools is effective in increasing vaccination rates in Puerto Rico?
    Probe: What is your opinion about this requirement? What are the pros and cons?
    Probe: How do you consider the quality (content) of the materials to notify about this requirement for the 2021-2022 school year?
        Probe: *Ex.* Circular letter of vaccination requirements for the 2021-2022 school year.
        Probe: How does the letter from this year 2021-2022 compare to the letter from 2020-2021 school year regarding the HPV vaccine dose requirement? Would you make any changes?

4. **Do you think there is any advantage to having this HPV vaccine requirement in schools compared to other strategies?**
        Probe: For example, an educational strategy to motivate vaccination.

COVID-19 SECTION

5. **Do you understand that it is necessary to include the HPV vaccine as a requirement in schools for adolescents between the ages 11 to 16 years?**
    Probe: How do you compare the priority given to HPV vaccine efforts (vaccine and the requirement) with COVID-19 efforts?
    Probe: Do you think that just like the HPV vaccine, the COVID-19 vaccine should also be added as a requirement for school entry?

6. **Organizational challenges:**
    For Department of Health employees: How complicated do you find it for the Department of Health to comply with this school requirement (HPV) currently?
    For school nurses/admin: How complicated do you find it for your school to comply with this school requirement (HPV) currently?
    For community-based organizations: How complicated do you find it for your organization to comply with this school requirement (HPV) currently?
    For medical providers: How complicated do you find it for your organization/clinic to comply with this school entry requirement (HPV) currently?
    Probe: Has the COVID-19 pandemic or any other event affected the execution of this requirement? How?
    Probe: Do you have any strategy in your program/department to ensure the required doses are completed for the next school year 2021-2022?
        Probe: Ex. Educational campaign, policy development, community efforts, education directed to parents.

For school nurses/admin: (ONLY FOR PRIVATE SCHOOLS) How has this process been in private schools? Has this process been different compared to public schools?

7. **Receptiveness to Implementing HPV Vaccine Requirement:**
For Department of Health employees: How receptive is the Department of Health in implementing this school requirement for the HPV vaccine during this pandemic?
For school nurses/admin: How receptive is the school where you work in implementing this school requirement for the HPV vaccine during this pandemic?
For community-based organizations: How receptive is your organization in implementing this school requirement for the HPV vaccine during this pandemic?
For medical providers: How receptive is the clinic where you work to implementing this school requirement for the HPV vaccine during this pandemic?
Probe**:** How do you think the collaboration has been between health professionals (doctors, nurses) and education professionals (teachers, principals) when reopening schools in your area?
Probe**:** Has the work environment at your job changed regarding vaccine implementation after the pandemic?
Probe**:** Do you have administrative support to implement this requirement?

8. **What modifications (adaptations) have you had to make in your organization due to the pandemic to comply with this school requirement (HPV)?**
Probe: Example: flexibility of requirement, extending requirement dates, providing the green paper (PVAC 3).
Probe: Have you had to reinforce these guidelines in your organization to comply with this HPV requirement during school reopening?
Probe: Has communication within your organization changed compared to the period before the pandemic?
Probe: Has communication with external organization changed compared to the period before the pandemic?

9. **How have parents of adolescents aged 11-16 reacted to this HPV vaccine requirement during this pandemic?**
Probe: What leve lof support or opposition exists from parents and their children currently?
Probe: Do you think the COVID-19 vaccine can influence parents to vaccinate their children against other infectious diseases such as HPV (including those who are against or undecided)?
Probe: What reasons do you believe parents have that keep them from vaccinating their children?

10. **Do you think parents/adolescents are aware of this requirement and understand the benefits of this HPV vaccine?**
Probe: Parents of young people aged 11-16.
Probe: Do you think the available information for parents and adolescents about this requirement and the vaccine is sufficient?
For Department of Health employees: Is the Department of Health providing or planning to provide information to the community about the HPV requirement and/or vaccine? (social media and other communication media).
For school nurses/admin: Is your school providing information to the community about the HPV requirement and/or vaccine? (social media and other communication media).
For community-based organizations: Is the Department of Health providing information to the community about the HPV requirement and/or vaccine? (social media and other communication media).

For medical providers: Is your clinic providing information to the community about the HPV requirement and/or vaccine? (social media and other communication media).
Probe: How easy or difficult is it for parents to obtain the vaccine?
Probe: What factors complicate obtaining the vaccine for parents?
Probe: What factors complicate completing the required doses?

11. What do you think about vaccine exemption policies?
Probe: Experiences (if any) with exemption requests.
Probe: How have exemptions increased/decreased compared to the period before the pandemic?
Probe: Reasons for requesting exemptions.

12. What type of resources (personnel and physical) are currently available for the implementation and monitoring of the HPV vaccine requirement in your program?
***Probe*:** How has the staff involved been trained to meet the requirement for the next school year?

13. Did your organization have to identify any budget to comply with this requirement for the next school year?
Probe: How expensive is it for your organization to carry out this requirement currently?

14. For the next school year, will you receive any help from an external organization, coalition, or individual that has formally influenced or facilitated the decisions of this school requirement (HPV)?
Probe: For example, community organizations, school-systems, pharmaceutical companies.

15. What recommendations do you think is needed regarding the policy or the HPV vaccine?
Probe: Principals, teachers, parents.
Probe: Department of Health personnel.

16. Specific Questions for Different Groups:
Department of Health (PREIS ONLY): How does the PREIS evaluate the implementation process?
Probe: How will the new electronic system help document vaccine administrations?
VTrckS Manager: How has the HPV vaccine inventory behaved compared to the period before the pandemic? Has it decreased, remained the same?

Interviewer: This concludes the question section. Do you have any questions about the project before we end the interview?

Interviewer: Thank you for your time. After we complete this phase of our study, we will analyze and review the key informant interviews with the information collected from the analysis of social media content and focus groups. This information will help us improve the procedures and implementation of the HPV vaccine public policy in Puerto Rico.
